# Specimen mass limitations undermine the analytic validity of quantitative tissue cultures

**DOI:** 10.1128/jcm.00585-26

**Published:** 2026-08-21

**Authors:** Tess A. Karre

**Affiliations:** 1 Department of Pathology, Microbiology, and Immunology, University of Nebraska Medical Center 12284, Omaha, Nebraska, USA; Mayo Clinic, Rochester, Minnesota, USA

**Keywords:** wound culture, pre-analytic variables, specimen mass, quantitative tissue culture

## LETTER

Quantitative tissue culture has historically been used to estimate bacterial burden in wound and surgical specimens, applying interpretive thresholds, such as ≥10⁵ CFU/g, to distinguish infection from colonization (1–3). However, the analytic validity of this approach depends on adequate specimen mass for reproducible homogenization, dilution, and organism recovery. Quantitative tissue cultures are frequently ordered in contexts where specimen quantity is intrinsically limited, constraining the feasibility of meeting analytic requirements for reproducible quantitation. Limited data exist describing whether submitted specimens consistently meet these basic requirements.

As part of routine test menu review, a retrospective evaluation was performed of all specimens submitted for quantitative tissue culture at our institution over 18 months to assess specimen suitability for quantitative analysis in real-world practice (Table 1).

**TABLE 1:** Specimen weights and quantitative culture results for tissue specimens submitted for quantitative tissue culture^*a*^

Number	Weight (g)	Quantitative result	Qualitative/quantitative culture concordance
1	0.030	No growth	Concordant
2	0.030	No growth	Concordant
3	Not recorded	No growth	Concordant
4	0.016	≥10⁵ CFU/g *Staphylococcus aureus*	Concordant
5	0.050	No growth	Concordant
6	0.014	No growth	Concordant
7	0.016	No growth	Concordant
8	0.007	No growth	Concordant
9	0.024	≥10⁵ CFU/g *Peptoniphilus harei*	Discordant (no anaerobes on concurrent anaerobic culture)
10	0.026	No growth	Concordant
11	0.015	No growth	Concordant
12	0.030	≥10⁵ CFU/g Group B *Streptococcus*; ≥10⁵ CFU/g anaerobes	Concordant

^*a*^ Quantitative culture results were interpreted using a ≥10⁵ CFU/g reporting threshold. All specimens were processed using the institutional quantitative tissue culture protocol.

Twelve specimens were submitted during this period with weight recorded for 11. The mean weight was 0.023 g (median, 0.024 g; range, 0.007–0.05 g). All specimens weighed <0.1 g, with most clustering between 0.01 and 0.03 g. Across the observed weight range, classification as ≥10⁵ CFU/g frequently depended on recovery of a single colony at commonly plated dilutions, underscoring the sensitivity of quantitative interpretation to minor analytic variation. Using the protocol employed in this study (2 mL homogenization volume with plating of 0.1 mL dilution aliquots), the calculated contribution of a single colony is inversely proportional to specimen mass and increases with dilution factor. For a 0.024 g specimen, a single colony recovered from the 10⁻² dilution corresponds to approximately 8.3 × 10⁴ CFU/g, approaching the 10⁵ CFU/g interpretive threshold. For smaller specimens or when higher dilutions are used, a single colony may exceed the threshold, such that classification depends on recovery of one colony or no colonies. Consequently, tissue weights in this range are unlikely to support robust discrimination around the 10⁵ CFU/g interpretive threshold despite the appearance of numeric precision.

In one instance, quantitative results differed from concurrent qualitative anaerobic culture findings, illustrating the potential for misleading interpretation when quantitative assays are applied under specimen conditions that do not support reproducible quantitation.

These observations align with current microbiology literature and reference guidance (2–4). While early studies suggested that bacterial burdens above defined thresholds correlated with impaired wound healing (1), subsequent literature demonstrated inconsistent associations with clinical outcomes and limited reproducibility (5, 6). Although authoritative references advise against routine use of quantitative tissue cultures, these methods are still described as valid under limited circumstances, which may contribute to their continued use in practice (3, 4).

The findings highlight a disconnect between the theoretical framework supporting quantitative tissue culture and the realities of specimen submission in practice. While quantitative tissue cultures may retain analytic validity under controlled conditions with sufficient specimen mass, the data demonstrate that, in routine clinical use, specimens frequently lack the mass necessary for reliable interpretation. Thus, our institution discontinued quantitative tissue cultures. Laboratories should re-evaluate the utility of quantitative tissue cultures considering specimen suitability and analytic variability. Qualitative deep tissue cultures with appropriate organism workup and susceptibility testing may provide more reliable and clinically actionable information.
